# Motivation and incentives of rural maternal and neonatal health care providers: a comparison of qualitative findings from Burkina Faso, Ghana and Tanzania

**DOI:** 10.1186/1472-6963-13-149

**Published:** 2013-04-25

**Authors:** Helen Prytherch, Moubassira Kagoné, Gifty A Aninanya, John E Williams, Deodatus CV Kakoko, Melkidezek T Leshabari, Maurice Yé, Michael Marx, Rainer Sauerborn

**Affiliations:** 1 Institute of Public Health, University of Heidelberg, Im Neuenheimer Feld 324, Heidelberg, 69120, Germany; 2 Nouna Health Research Centre (CRSN), Nouna, Burkina Faso; 3 Navrongo Health Research Centre, Navrongo, Ghana; 4 School of Public Health and Social Sciences, Muhimbili University of Health and Allied Sciences, Dar es Salaam, Tanzania

**Keywords:** Health personnel, Motivation, Incentives, Maternal-Child health services, Developing countries

## Abstract

**Background:**

In Burkina Faso, Ghana and Tanzania strong efforts are being made to improve the quality of maternal and neonatal health (MNH) care. However, progress is impeded by challenges, especially in the area of human resources. All three countries are striving not only to scale up the number of available health staff, but also to improve performance by raising skill levels and enhancing provider motivation.

**Methods:**

In-depth interviews were used to explore MNH provider views about motivation and incentives at primary care level in rural Burkina Faso, Ghana and Tanzania. Interviews were held with 25 MNH providers, 8 facility and district managers, and 2 policy-makers in each country.

**Results:**

Across the three countries some differences were found in the reasons why people became health workers. Commitment to remaining a health worker was generally high. The readiness to remain at a rural facility was far less, although in all settings there were some providers that were willing to stay. In Burkina Faso it appeared to be particularly difficult to recruit female MNH providers to rural areas. There were indications that MNH providers in all the settings sometimes failed to treat their patients well. This was shown to be interlinked with differences in how the term ‘motivation’ was understood, and in the views held about remuneration and the status of rural health work. Job satisfaction was shown to be quite high, and was particularly linked to community appreciation. With some important exceptions, there was a strong level of agreement regarding the financial and non-financial incentives that were suggested by these providers, but there were clear country preferences as to whether incentives should be for individuals or teams.

**Conclusions:**

Understandings of the terms and concepts pertaining to motivation differed between the three countries. The findings from Burkina Faso underline the importance of gender-sensitive health workforce planning. The training that all levels of MNH providers receive in professional ethics, and the way this is reinforced in practice require closer attention. The differences in the findings across the three settings underscore the importance of in-depth country-level research to tailor the development of incentives schemes.

## Background

Across Sub-Saharan Africa progress in the reduction of maternal and neonatal deaths remains insufficient. It is increasingly recognised that this is both caused by, and reflects, weaknesses in entire health systems [1,2]. In Tanzania in East Africa, and Burkina Faso and Ghana in West Africa the maternal mortality ratios are estimated to be 790, 560 and 350 per 100,000 live births respectively [3]. The figure is lowest in Ghana, which is also the only country of the three that is making progress towards attainment of the fifth Millennium Development Goal (MDG). Neonatal death rates are around 30 per 1000 live births across the three countries [4]. Poverty levels vary across the countries: Ghana is a low middle-income country with a gross national income (GNI) per capita of $1,230. Tanzania and Burkina Faso are both low income countries with GNI per capita of $530 and $550 respectively [5]. Approximately 83% of the population of Burkina Faso is classified as poor using the multidimensional poverty index, compared to 66% in Tanzania and 30% in Ghana [6].

All three countries are engaged in efforts to improve maternal and neonatal health (MNH) care. These include measures to raise the number of facility-based deliveries and increase the availability of Emergency Obstetric Care [7-9]. However, these measures and the overall quality of MNH care are beset by challenges, many of them human resources related. All three countries face critical shortages of skilled health staff. The most recent WHO estimates for the number of nurses and midwives per 10,000 population are 9.8 in Ghana, 7.3 in Burkina Faso and 2.4 in Tanzania [10]. Moreover, in all three countries the distribution of such staff is skewed towards urban areas, although in Ghana just over half, and in Tanzania and Burkina Faso the majority of the population continue to reside in rural areas [11]. Medical doctors are even scarcer in rural areas, with many preferring to work in urban areas at higher level facilities, in the private for profit sector or in other countries [12]. The reasons for the shortages of human resources for health are many and include the brain drain. The estimated expatriation rate in 2000 for medical doctors born in Ghana but working in the OECD countries was 31.2% and 24.9% for nurses, compared to 55.3% for doctors and 6.8% for nurses from Tanzania, and 7.6% for medical doctors and 0.3% for nurses from Burkina Faso [13]. Data capturing the regional brain drain, for example, of health staff from Tanzania to Botswana or South Africa [14], or from Burkina Faso to Cote d’Ivoire [15] is less readily available.

All three countries are working to scale up the numbers of available staff, as well as to improve skills levels. Moreover, there is recognition of the need to address the issue of health worker motivation. There is general agreement regarding the benefits associated with a motivated health workforce; motivated staff are more likely to apply their knowledge to the actual delivery of clinical care, minimizing the so-called ‘know-do gap’ [16,17]. Luoma (2006, p.3) observes that ‘enhanced worker motivation leads to improved performance’ [18]. Policy attention in these three countries, and beyond, is focused upon how the motivation of health workers in low-resource settings can be improved, including through the introduction of incentives, and there is an interest to expand the knowledge base in this regard.

The work of Franco et al. [19] offers a useful point of reference for studies into motivation in developing countries [19]. Their conceptual framework facilitates a broad exploration of the different levels that can affect health worker motivation - spanning policy, organisational, community and individual level influences. This multi-level approach underpins the current study which explores MNH provider views about motivation and incentives at primary care level in rural Burkina Faso and offers some comparisons to views amongst the same group of providers in Ghana and, to a lesser extent, in Tanzania. The findings from Tanzania have been reported upon separately [20].

Involvement in this study was based, at the institutional level, on long-standing relations and cooperation. However, the final selection of the three countries in Sub-Saharan Africa was ultimately made to facilitate comparison: They span the East and West of the continent and have distinct ancient and recent histories, although these cannot be explored in detail here. Burkina Faso uses French as the official language, Ghana uses English and in Tanzania Kiswahili has been established as the national language. Moreover, the countries have varying levels of poverty, are distinctly affected by the brain drain and are making different levels of progress towards MDG 5.

In all countries the study area comprises remote, disadvantaged and predominately rural districts - Lindi Rural and Mtwara Rural in the South of Tanzania, Nouna and Solenzo in the North-West of Burkina Faso and Builsa and Kassena-Nankana in North-Eastern Ghana.

This study was conducted in the frame of the QUALMAT research project which seeks to improve the quality of MNH care. It was undertaken to inform work package 5 which explores the role of provider motivation in the quality of such care. The QUALMAT project will, amongst other interventions, pilot incentive schemes at rural primary level facilities. The main research questions were: which factors motivated these respondents to join the health professions; what is understood by the term motivation; what influences their motivation, job satisfaction and the quality of their care; which incentives do these providers themselves suggest.

## Methods

The concept of motivation has been shown to be closely intertwined with socio-cultural context [21]. Therefore qualitative research was considered to be essential to address the research questions. In-depth interviews were the methodology of choice. Respondents were the providers of maternal and neonatal care at primary care level and their managers at facility, district and national level. The selection of respondents from all levels of the health system was undertaken in reference to the multi-level framework of Franco et al. [19]. The process of elaborating the topic guide is described in detail with the Tanzania findings [20]. In short, a draft was prepared based upon the literature and the authors’ previous experience. Care was taken to include questions relating to all the levels of possible influence suggested by Franco et al. [19]. The draft was further developed in a participatory process involving psychologists, social scientists and health workers from the three countries concerned. Experienced, local, qualitative researchers were selected to take the interviews. The topic guide was kept quite structured and a streamlined methodology preferred because of the interest to gain an impression of the similarities or differences between the findings generated across the three settings.

A series of meetings were arranged to make it possible for the research team to work together despite being based in different countries and communication being both in English and French. At the first, pretest results were reviewed and the topic guide finalized in English. Consensus was reached on how the interviews would be approached. Moreover, decisions were taken to record the interviews if the respondents agreed and, in the case of Tanzania, to translate from Kiswahlili to English during transcribing. An introduction was given to the software NVivo9® as a tool to facilitate the coding and analysis process (QSR International Pty Ltd, 2007). This software was already in use at two of the institutions involved meaning support could be provided. At this stage a main coder (first author), with previous experience and familiarity with all the languages involved, was identified. It was further agreed that the main coder, being highly familiar with the Tanzanian context, would undertake preliminary coding of that data set and the further coding rounds for all three data sets. Preliminary coding for the Burkina Faso and Ghana data sets would be undertaken by the interviewers themselves.

After the first meeting, the topic guide was translated into Kiswahili and French. The back translation method and pre-tests were used to ensure quality. A short instruction document was drawn up for use in all the settings. The interviewers then set about generating the data. In each country the sampling framework was purposive, focusing on the intervention and non-intervention districts of the QUALMAT study, and on primary level facilities. Health centres with a maternity unit and located over 10 km from a town were defined as rural. A total of 35 interviews were held in each country with 2 policy level informants, 4 district and 4 facility level managers and 25 health providers involved in delivering MNH at PHC level. Given that the human resources situation is known to be particularly poor in primary facilities in rural areas in all the countries the emphasis was put upon the staff found to be delivering MNH on the ground and not upon their profession. From now on these will be referred to as ‘MNH providers’. In each country 5 MNH providers from the private sector were also included to capture their views. In Tanzania and Ghana these respondents came from faith-based facilities. In Burkina Faso there were no private facilities in the study districts, so these respondents were sought from a private for profit facility located in Bobo-Dioulasso. The sample size was decided in keeping with the intention to gain an in-depth insight into provider views and perceptions [22]. Across all the countries the interviews took place between June-July 2010. They were pre-arranged and conducted away from the work environment after duty hours, taking approximately 2 hours each. The interviewers transcribed the data themselves to maximize accuracy. All those agreeing to take part signed consent forms. Willingness to take part in the study was high, with none of those approached refusing to take part, and only one respondent in Burkina Faso and one in Ghana preferring notes to be taken instead of the interview being recorded.

At the second meeting time was spent to read through and reflect upon the transcripts. These were then uploaded into a single database separated into three country datasets. After a discussion on the general approach, the process of preliminary coding was commenced. NVivo was then used to undertake inter-coder reliability testing. All the responses to selected segments of the topic guide from Burkina Faso, and afterwards from Ghana, were coded by the main coder and the county interviewer. In both cases the results were compared and any areas of disagreement jointly examined. This process was continued until Cohen’s kappa scores were in the range of 0.7 or above. This score indicates substantial levels of agreement [23].

The preliminary coding was completed after the researchers returned home, the data sets uploaded from the different sites and then inserted back into the main database. The next coding stages were all carried out by the main coder to avoid further inter-coder reliability issues. These involved working systematically through each country data set, looking for unprompted comments to the various topics and then ‘cross coding’ them to all the locations where they fitted thematically. Next in-depth analysis was undertaken; the themes were broken down and cross-cutting issues and patterns sought and discussed in country-teams. Finally, the three datasets were merged together and a last round of cross-country analysis undertaken. During the process of writing up the findings the main coder translated the quotes from Burkina Faso from French to English. Statements that are indicative of general tendencies in the responses have largely been selected for quotation.

Ethical clearance was granted by the Institutional Review Board at the Navrongo Health Research Centre (ID NHRCIRB 085) for Ghana; the Muhimbili University of Health and Allied Sciences Ethical Review Committee (ref no. MU/AEC/VOLXIII/96) for Tanzania and the Ethics Committee for Health Research for Burkina Faso (ref. 2010.05/CLE/CRSN).

## Results

The type of providers found to be delivering MNH care at primary level in rural areas across the three countries varied. In Ghana the providers were largely midwives, nurse-midwives or community health nurses. In Tanzania, most fell into the categories of nurse-midwife, nurse or nurse attendant. An overview of the respondents from Burkina Faso is provided in Table 1. In Ghana and Tanzania most MNH providers were female, in Burkina Faso 13 of the 25 MNH providers were female and 12 were male. An important proportion of the MNH providers were made up of lower cadres in Tanzania (nurse attendants) and Burkina Faso (auxiliary midwives). In all three countries women were under-represented at the managerial levels, particularly so in Burkina Faso.

**Table 1:** Profile of the in-depth interview respondents from Burkina Faso

		**Function**				**Sector**		**Age in years**				**Sex**	
**Profession**	**Description**	**Policy maker**	**District manager**	**Facility manager**	**Health worker**	**Public**	**Private**	**20-29**	**30-39**	**40-49**	**50-59**	**Female**	**Male**
Human Resources Advisor	One of the policy level respondents had a none-health background which was not further explored.	1				1					1		1
Medical Doctor	A level education (7 years of secondary school), plus 6 years of university level training and 1 year internship.		3			3			3				3
Registered Nurse (Infirmier Diplômé d’Etat)	2 years pre-service training open to those who have completed 7 years of secondary education in receipt of a certificate.			4	8	8	4	2	9	1		1	11
Midwife (Sage-femme)	3 years pre-service training open to those who have completed 7 years of secondary education. (Auxiliary midwives can also upgrade by undertaking 2 years more training).	1			4	4	1	1	1	1	2	5	
Male Midwife (Maïeuticien d’Etat)	3 years pre-service training open to those who have completed 7 years of secondary education.		1		4	5		2	1	2			5
Auxiliary Midwife (Accoucheuse Auxiliaire)	2 years pre-service training open to those who have completed 4 years of secondary education.				8	8		3	3	2		8	
Community Outreach Worker (Agent Itinérant de Santé)	2 years pre-service training open to those who have completed 4 years of primary education.				1	1			1				1
Totals		2	4	4	25	30	5	8	18	6	3	14	21

### Choice of, and commitment to, profession and rural workplace

In all three countries, many described being drawn to their profession by a sense of vocation. In Burkina Faso there were also widely held views that health work was honourable and that a good health worker would leave a positive legacy and be well remembered. A very strong sense of duty was attached to helping ones’ fellow Burkinabes. Some respondents reported joining their profession to be able to improve the health of their family and neighbours. Concurrently, frequent mention was made of the importance of having secure employment, which the policy level respondents considered to be an influence of ever increasing importance. Several very explicit and pointed references were made that the salary had *not* been a reason for becoming a health worker.

Such strong sentiments of health work being a duty, almost a moral obligation, were echoed in the views of providers working in the faith-based sector in Tanzania, *“It is my duty to help the poor. It is here where the mission works that the community is in need. When joining we were asked to write a letter saying why we would like to become a nurse. I wrote that this is a good job, a way to serve other people and God”* (Nurse 2, female, faith-based facility, Tanzania).

In Ghana the wish to serve one’s people was not only cited as a reason for joining the profession, but also as a reason not to emigrate. Moreover, in contrast to the responses from Tanzania, considerable admiration and status was attached to being a health professional. The responses from all three settings indicated that professional expectations were more likely to have been met amongst those with a clearer understanding of health work, due to a relative or acquaintance already being engaged in such a role.

Across the three countries most of the respondents described having been assigned to, rather than having chosen, their rural posts. Rural posts were associated with problems. In Burkina Faso and Tanzania these included the availability and cost of water and electricity, the distance to one’s family and being poorly informed about new developments in the health sector. The respondents in Burkina Faso considered living conditions in rural areas to be greatly inferior to those in urban areas. The responses from Ghana indicated that although many providers had staff housing, there was resentment about the size, state of repair or furnishings, and whether the houses were in keeping with their positions. Rural facilities were generally portrayed as being out of touch, unfashionable and offering few opportunities, *“There are so many problems here in the village. You can even see that the colleagues in town look better than you. Look at my hair-style. I don’t resemble those smart colleagues in town. That is the truth I am telling you. Sometimes you will meet colleagues in town and they will ask what you are doing there and if there is nothing to do at the health centre. It is like they think you are lazy, or even that you don’t have the right to go to town” *(Midwife 5, female, public facility, Ghana).

Commitment to remaining a health professional was generally high across all three countries. Commitment to rural posts seemed strongest in Tanzania, despite the complaints. In Burkina Faso this commitment appeared to be time-bound, with frequent reference made to staff turn-over. The majority of the respondents were in the age range 20–39 years, with very few older staff. Three of the four district level respondents from Burkina Faso were medical doctors. All of them referred to better working conditions in neighbouring countries and reported that they would consider emigrating.

Many of the respondents from Ghana indicated that they would prefer to leave for an urban position. The strong sense of being professionals and wanting to get ahead fitted uneasily with rural postings which were perceived to lack incentives and status, “*There is no motivation to stay in rural areas, none at all. We are supposed to get something for the deliveries but the amount is very negligible. For now it is 10% of the delivery cost, about GH¢9.90 and 10% of that is less than GH¢1 which cannot even buy a bottle of malt [fizzy drink*]” (Midwife 8, female, public facility, Ghana). Several respondents described feeling forgotten and there were reports of some health workers having taken to drinking. None of the respondents mentioned planning to shift to the private sector, but several stated that opportunities to engage in additional work at private health institutions were easier to come by in towns. Whilst the policy level respondents talked about health workers from Ghana seeking to go abroad, the respondents from the other levels of the health system expressed no such intent. In all three settings there were some providers who demonstrated strong commitment to their rural posting. Most of them originated from the area, or appeared to have made a decision to settle there.

### Understandings of motivation

In Burkina Faso motivation was described as follows, *“When I think of motivation I think of stimulation. It is a thrust to act, a drive that pushes you ahead. Motivation can be financial, verbal or moral. Without this thrust you cannot be efficient and get results”* (Registered Nurse 2, male, public facility, Burkina Faso). Several specifically stressed the non-financial nature of their understanding. When referring to their own motivation, most of the respondents described themselves as being fairly motivated. The staff that mentioned having entered the professions to be useful to their own relatives and neighbours described themselves as being well motivated.

In Tanzania, the understandings of motivation were mixed. Whilst some respondents referred to aspects such as the motivating effect of appreciation and praise, the overall, dominant theme was of motivation being a positive incentive given over and above ones salary [20]. Similarly amongst the responses from Ghana, the understanding of motivation was generally of it being something external that was done or given, and often of it being something tangible. *“Motivation is about giving someone something extra for good work done. It shows that it is noticed when they have worked late and spent less time with their family. If at the end of the month we get something outside your salary, it will motivate us more to work harder”* (Midwife 2, female, public facility, Ghana).

The responses from Ghana also made reference to the importance of health workers having ‘an inner drive’, but this was not perceived to be part of motivation, *“I would say that we are not motivated. Our salary is too low for that. As a health worker a fellow must have the inner courage that this is what he want to do to keep going despite the challenges* (Facility In-charge, Medical Assistant 1, Male, public facility, Ghana).

Glimpses of more intrinsic dimensions to the understandings were also revealed *“My motivation is actually low. In fact there is none. But my in-charge, she will always say ‘today you have done well’ which in itself encourages me to work harder”* (Midwife 9, female, public facility, Ghana). *“I think my motivation is fair because of my clients. They appreciate what I do for them, some come to thank me, others pray and ask God to give me blessings. That one is my real source of motivation”* (Midwife 4, female, public facility, Ghana)*.* However, overall the responses from Ghana and Tanzania generally described MNH provider motivation as being fair or low.

### Job satisfaction

Across all three countries the respondents indicated that MNH providers experienced a reasonable degree of job satisfaction. The reasons for this were largely intrinsic, most notably client appreciation. The providers in Burkina Faso who had remained in a rural area for a long time described themselves as being very satisfied, *“I can say yes, I am extremely satisfied because I am from here and I am used to these people. I have a close collaboration with the mothers and their children. Now, those babies to whom I gave life have grown up. They are now coming here to deliver their own children, and this is a* pretty sight” (Midwife 3, female, public facility, Burkina Faso).

The responses from Ghana showed that, beyond client appreciation, job satisfaction was derived from the pride MNH providers took in their work, as well as appreciation from managers and good team relations. Across the three countries, the providers reported their managers taking only a limited interest in their satisfaction. Moreover, the policy level respondents all considered MNH providers to experience only low levels of job satisfaction.

### Working conditions

Across all the three countries, working conditions were described as difficult and the MNH workload as particularly heavy, in part due to the lack of staff that were trained in this area. The specific challenges of caring for neonates were only mentioned in Ghana. The responses from the other countries focused very much on the maternal aspects. Moreover, strong concerns about the occupational risks of their work spanned the countries, as did appreciation of any steps taken to address them.

In Burkina Faso, despite the many references to being part of a team, other health staff were portrayed as being reluctant to become too involved in MNH tasks and the complaints about MNH workload stemmed predominately from female midwives and auxiliary midwives. In Ghana, divisions of labour were shown to be strong, with the different professions prescribed precise roles. In Tanzania the facility managers described how under-qualified staff were being used to fill gaps in the delivery of MNH care [20]. In the responses from Tanzania, and to a lesser extent Ghana, the more highly skilled complained about the habits of other staff and the mistakes they made. The providers who felt competent in MNH made it very clear that whilst they could step in for other health workers no-one could step in for them.

### Management

In Burkina Faso the role of facility managers appeared to be facilitated by a strong sense of discipline and work ethic, “*All of us Burkinabe, first we are workers. Even our farmers rejoice at their work in the field. So when my superior puts their trust in me then I must answer to that trust. I must work hard and not cause disappointment”* (Midwife 1, male, public facility, Burkina Faso). The views of managers appeared also to be broadly respected and their appreciation was considered encouraging, as was being given more responsibility.

Little attention was afforded to the appraisal process or to job descriptions. Several respondents reported being in their posts for close to 2 years and not yet having been appraised. Job descriptions appeared to generic and were not considered to match the actual demands made*.* Despite the reported high levels of staff turnover, good relations between staff were repeatedly mentioned, *“I feel comfortable working here…….. In most instances I can rely on my experience. But if I am not sure then I do not worry but ask my colleagues for their help”* (Auxiliary Midwife 6, female, public facility, Burkina Faso). Supervision carried out well by those from higher up the system was considered very encouraging. However, most of the respondents resented the infrequency and haste with which district staff undertook supervision at facility level.

In Tanzania the responses showed the position of facility managers to be highly challenging. Many of the providers considered their managers to be unsupportive and attached little value to their feedback [20]. This may reflect on the failings described in the processes of supervision and performance appraisal, both of which were considered discouraging, as was being given more responsibility. Several respondents described difficulties in getting all the team members together to conduct supervision. There were references from older respondents from both public but particularly faith-based facilities that discipline was no longer as it used to be, *“When I trained we obeyed the matron. It didn’t even need to be said. If they told us to do a task then we got on and we did it. Now people answer back and complain. Even if they say yes, later you can find that the task is still not done. Some are even rude and refuse outright”* (Nurse-midwife 4, female, faith-based facility, Tanzania).

From the responses in Ghana it emerged that winning the approval of managers was extremely important, not least for getting a good appraisal and future promotion. The interviews were marked by a strong sense of competition, *“I just focus on doing my work right. Everywhere I work this is what I do. I was once give the prize for the best health worker in the region. Of course this makes the other staff envious and they talk badly about me, they mock….[pauses]..I have been through that but I don’t listen. In the end that sort of human jealousy cannot actually harm us”* (Midwife 4, female, public facility, Ghana). Situations were revealed where staff preferred to keep quiet if they were unsure, *“If you don’t know something it is better just to try and solve it on your own. If you ask the others they can just laugh. They ask where you went to school or tell you to improvise. So I stopped asking. There is no way they will help you”* (Enrolled Nurse 1, female, public sector, Ghana).

Some of the providers mentioned having managers that worked alongside them and described this as extremely encouraging. However, not all managers were referred to with respect. There were criticisms about them using their position to avoid hard work, of their indulging in favouritism or being frequently absent. Almost all the providers reported discouraging experiences of award ceremonies, describing how the ‘wrong’ staff were chosen and decorated.

### Community relations

In Burkina Faso some facilities were reported to be well used but others were not. There were examples of poor communications, and a language barrier, between providers and the Dioula-speaking communities, *“Sometimes we have dealings with patients who do not have a good understanding of the processes here. When it comes to discussions about referral or payments it can be very difficult. They can also make associations that are not correct – they may think ‘this is the one who injected my child and then it died’. As providers we have to explain things very well to the people so they can understand”* (Midwife 4, male, public facility, Burkina Faso). References to the language barrier also marked the interviews from Ghana. Across all three countries there were indications of social distance between communities and staff, including descriptions of younger staff from different parts of the country failing to show sufficient respect to older patients.

This notwithstanding, appreciation from the community encouraged the respondents from all three settings, *“Seeing those who came for services again and hearing them say thank you goes straight to the heart. I remember a woman thanking me for helping her deliver. This woman had very little but she gave me some eggs. It was really motivating to be appreciated so warmly”* (Auxiliary Midwife 2, female, public facility, Burkina Faso).

### Anxieties at work

In Burkina Faso anxieties included concerns about being separated from ones family, inability to treat some patients, as well as being bribed to favour certain patients over others. It was a source of extreme anxiety to some of the providers that they suspected their facility manager of being involved in financial corruption. Moreover, some male providers had concerns about working in MNH in conservative, rural areas. *“Once I was attacked by the husband of a patient who believed I had assaulted her. This really affected me. You often find yourself alone in front of women in this job and there are often situations that are uncomfortable”* (Midwife 1, male, public facility, Burkina Faso).

In Tanzania the anxieties also included workload, as well as being blamed by the community for issues that were out of the providers control - even being accused of theft when supplies ran out - and being overlooked for seminars. In Ghana they concerned favouritism, when superiors abused their power, infrequency of promotions, suspicions between team members as to how hard people work, and being left to work at a facility completely on one’s own.

Across the responses from all the countries, many of the providers were shown to be uncertain about who to turn to regarding such concerns.

### Remuneration

In Burkina Faso a proportion of the respondents, particularly females and lower cadre workers, showed a degree of acceptance of their salary. Many described earning enough to cover their basic costs and appeared to put their wage in the context of the country’s general economic situation *“Well, I think it's better to be honest given the problems facing Burkina. I am satisfied with what I receive at the end of the month. I find it acceptable, since I manage to solve my problems with this salary” *(Auxiliary Midwife 13, female, public facility, Burkina Faso). There was also agreement that salary levels were transparent, that everyone knew how much the different cadres were paid and that salary payments were made on time. However, amongst the more senior respondents frustration was revealed, “*Is this even worth talking about? There will be no change until after we have died. The government doesn’t listen and can’t do anything. In Burkina we are simply poorly paid”* (District manager, medical doctor 1, male, Burkina Faso).

The respondents from Tanzania and Ghana all complained repeatedly that their salary was too low and not in accordance with the level of responsibility or volume of work that was expected. The respondents of both countries expressed concerns about the intransparency of salaries and there were suspicions that other colleagues might be earning more. The facility in-charges from Tanzania complained that their additional managerial function was not properly remunerated.

When asked about the link between salary levels and performance, the respondents in Burkina Faso described that it was up to their superiors to make this link by acknowledging when they worked well. The responses from Tanzania and Ghana considered the link to be missing, according it greater attention, “*The Government should set aside a fund just to motivate health workers. It is not your salary because that goes with the job and you are sure of it every month. Besides, I may be working hard and another may be lazing about and at the end of the month, we collect the same salary”* (Community Health Officer 2, female, public facility, Ghana). Indeed, a view emerged that some providers expected to receive something additional to their salary to actually work well.

### Quality of care and performance

In Burkina Faso a theme that emerged amongst the references to quality was that of the ideal and reality. There was a strongly held view that what was written in policy documents did not apply to rural realities, *“I will not return to the definition given in the health schools there eh! This is care given by highly skilled health workers who work under flawless conditions. Is this what we have here? No! In all the papers it is the same. The definitions are like a song. We find it everywhere. But here it is no-where, there is nothing like it”* (Facility manager, Registered Nurse 5, male, Burkina Faso).

Across all the countries the responses showed the MNH providers to be very clear that the goal of their facility was to deliver care to the community and there was much agreement that community use of services was a measure of quality. Respondents in all the countries agreed that in rural areas one’s level of competence was quickly exposed and that this effect was magnified in the area of MNH [20].

### Interpersonal relations

In Burkina Faso, beyond frequent reference to serving the community and the anxiety caused when patients could not be helped due to failings of the health system, indications emerged that the providers themselves did not always treat patients to the best of their ability. Several providers showed frustration with certain community behaviours “*These women insist to try and deliver at home. This is something that we discuss at village meetings. Yet still it happens. They only come here when things go wrong. In such cases I do not hesitate to scold them”* (Auxiliary midwife 4, female, public facility, Burkina Faso). Repeated mention was made of there being ‘other colleagues’ whose main interest was not to treat patients but rather financial, *“There are health workers whose primary objective is to make money. Compare this to health workers with a sense of vocation who will do anything to improve the quality of care. This has effects on the professionalism throughout”* (District manager, medical doctor 3, male, Burkina Faso). It was further mentioned that when providers came under financial pressure, for example, when loans had to be repaid, then such tendencies could take an upper hand. There were also hints that frustrated health workers might take things out on the patients. “*If the colleague is struggling to meet some costs and his work is heavy and the roof of his house is leaking, then all of this will play on his work. Someone who is angry all the time about things that are out of reach….this person can pour his anger on the patients! He will not greet kindly. He does not even care whether the treatment has any effect. At this time he does not even want to work”* (Facility manager, Registered nurse 8, male, public facility, Burkina Faso). This may be reinforced by the view that health workers were entitled to respect from patients, which seemed to be understood as obedience. “*There are times when you will ask a woman to be quiet, but if she persists you might give her a little slap to remind her to do as you say. To remind her that it does not make a good impression to others who hear screams come from the facility”* (Auxiliary midwife 7, female, public facility, Burkina Faso).

Reflections of similar views were also found amongst the responses from Tanzania; there were hints that providers overrode patient’s wishes when it came to the need for referral, and of patients being blamed inappropriately. There was talk of there being colleagues nowadays who lacked concern for the patients, *“One has the impression that these days people are becoming health workers for the money and to have a job. That love of the profession is no longer the same. They do not work for the welfare of maternal health so it is hard to admire them. It disappoints me a little”* (Enrolled Nurse Midwife 1, female, public facility, Tanzania). Several managers noted that to get things done one had to allocate the tasks to staff by name to make it clear that they were personally responsible.

In Ghana, there were repeated complaints that younger health workers were more interested in money and in competing with one another than in caring for the patients, *“You know, these young nurses are just interested in money. They are angry now that everything is free at the health facility for those who are insured, ANC, childbirth, child welfare side all the activities there are free. But some of them will still maneuver to take something small from clients”* (Midwife 4, female, public facility, Ghana). Some of the providers drew attention to the importance of their faith in not being drawn into such behaviours.

### Incentives

Across all three countries respondents suggested the following financial incentives: salary increase, (increased) rural allowance, greater possibilities to attend seminars, payment of overtime and leave, access to loans at good conditions and pension provision. In Burkina Faso the lack of a rural allowance was particularly mentioned, as were the demands for an increase in the housing allowance and for health insurance. In Ghana reinstatement of the additional hours allowance was highly sought after, as were improved benefits in retirement. Two of the private sector respondents were over the age of sixty and described having to continue to work for financial reasons. Several simply suggested that an extra cash payment be made to rural health workers on a monthly or quarterly basis. In Tanzania the faith-based respondents largely confined their suggestions to higher salaries, improved pension packages and access to seminars. Regarding the latter, there was a perception that public health workers were favoured with regards to such access. The benefits of seminars were closely linked to the payment of per diems causing them invariably to be referred to as financial incentives.

Across all three countries respondents suggested the following non-financial incentives: to increase the number of staff, enhance access to training and promotion, avail better equipment for health facilities, including for staff health and safety, improve housing and provide transport. In Burkina Faso there was additionally a strong suggestion from the public sector respondents to decorate good workers. In Tanzania, the call regarding housing was in fact for construction materials be given to health workers for them to build themselves. The request for support with transport also included reimbursement of travel costs to and from work if the lack of staff houses meant a provider had to rent in town. Others suggested that utility bills should be subsidized. None of these suggestions were mirrored in the policy level respondents ideas indicating a possible mismatch. A few respondents mentioned a certificate or letter of appreciation. In Ghana paid study leave was also highly sought after. In addition, supervision, flexible working hours and renovation of housing were suggested. Several mentioned that it would be helpful if health care benefits could be extended to all family members and into retirement.

In Burkina Faso there was overwhelming agreement that financial incentives should be given to entire teams. The majority also considered that non-financial incentives should be given to teams, with the exception of decorations. Despite the importance of the team, it was highly accepted that if someone did well then they should be decorated for this as an individual. In Tanzania most of the respondents were of the opinion that both financial and non-financial incentives should go to individual MNH providers. In Ghana no clear opinion emerges regarding this issue. However, strong concerns were raised about whether such schemes would be well and fairly managed. The Ghanaian policy makers highlighted how financial incentives needed to be quite significant to make a difference, and that the way such incentives proportionally relate to salary was important.

## Discussion

This study reaffirms the usefulness of Franco et al. (2002) framework for exploring health worker motivation at primary care level in low resource settings [19]. The findings demonstrate the complex interplay of influences on the motivation, quality of care and job satisfaction of rural MNH providers. They also serve to illustrate differences in views regarding preferred types and modalities of incentives between those at different levels of the health system, and between the three countries.

### Choice of profession and rural posting

The responses from Burkina Faso show that health work was perceived to be a noble undertaking, whilst those from Ghana associated the health professions with status and respect. The responses from Tanzania did not reflect these impressions. This could be because in Tanzania many of the respondents were mid or lower level cadres. Other studies from Tanzania report that such cadres command less respect from higher qualified staff and patients [24], and that patients low respect for health workers can even result in them being blamed for failings in the system [25]. Indeed, the fear of being wrongly blamed stood out as a key cause of workplace anxiety amongst the responses from Tanzania.

Regarding the rural workforce, the recruitment of female MNH providers emerged as a specific problem in this area of Burkina Faso, as has also been found in other conservative settings [26]. Reasons are likely to include the general challenges women face to do well at school or to forge a career in Burkinabe society [27]. In addition, cultural and security concerns appeared to surround the posting of single women away from home, or expecting a husband to relocate for his wife. The comparatively low numbers of female MNH providers is likely to play a role in the low reported use of some health centres. A review of 41 Demographic and Health Surveys revealed that one quarter of women cited the absence of a female health provider as a reason not to deliver at a facility [28].

Across all three countries, the concerns about rural postings partly transcend the health sector. For example, the state of roads and the availability of utilities like electricity. However, the view held by the respondents from Burkina Faso that accommodation was undoubtedly better in urban areas, and from those in Ghana that the state of housing and furnishing were not in keeping with the providers status could be addressed. This latter concern has also been noted by other studies conducted in Ghana [29,30]. The wish of those working at rural facilities to be kept abreast of new developments in the sector could also be tackled. Given the importance attached to the hierarchy that broadly spans all the responses, those from higher up the system such as members of the regional health management teams, could provide regular updates from the central level.

In all the three countries there were some health workers, often from the area in question, who expressed a readiness to remain in their rural posting. Community support has been shown to be crucial for making health workers feel welcome in such settings [31]. Failure to facilitate sufficient introduction may mean that only those actually originating from the area readily manage to achieve acceptance. As it has been shown that health professionals with a rural background are more likely to accept rural postings [32], the recruitment of students, especially girls, from local schools who are interested in a health career should be encouraged. There is also a need to explore more closely the incentives that these particular health workers prefer, such as payment of utility bills, access to loans at good conditions to facilitate home building, or the receipt of building materials in kind. The latter would have the additional advantage of further binding such health workers to the area. These proposals were all notably absent from the policy level suggestions of incentives. Others have also drawn attention to the lack of coherence between retention measures and an analysis of the preferences of health workers themselves [32].

More attention could be also be paid to recruiting the ‘right’ health workers, as some faith-based institutions in Tanzania appear to do routinely by asking for a letter of motivation. It is noteworthy that the respondents from Burkina Faso who reported having become health workers for the benefit of those in their immediate vicinity were found to be well motivated. Leon and Kolstad (2010) observed that Tanzanian medical students who joined the profession out of an interest to learn about health were more likely to remain motivated during their training [33].

It was realized retrospectively that such providers were assumed to have been well informed when they chose to become a health profession. However, Kijo (2011) highlight the lack of support and guidance students in Tanzania received from either schools or parents when selecting their future area of study [34]. Orenuga and da Costa ([35] p.996) argued that career choice in Nigeria strongly relates to ‘the image of a profession as a vehicle for the achievement of personal goals’ [35]. School-leavers in Tanzania were found to want to study medicine for similar reasons, whilst their perceptions of nursing were less favourable [36]. That the expectations of the respondents who were better informed about what health work actually entails were more likely to have been met in practice is an important finding in this regard. There is scope for further research in this area.

### Understandings of motivation

In Burkina Faso motivation was understood to be a multi-faceted concept, spanning financial, verbal (appreciation) and moral, intrinsic aspects. It was repeatedly described as being a thrust or ‘inner drive’ which alludes to the way motivation itself cannot be seen [37]. In particular, strong reference was made to ‘moral motivation’ which may link with the nobleness ascribed to health work and the widely mentioned need for health workers to make sacrifices. The responses from Burkina Faso indicated that the MNH providers were fairly motivated, which would tie in with this understanding.

In Ghana and Tanzania the understandings were more mixed. When asked to define motivation there was a strong focus on it being a tangible incentive - something given in addition to one’s salary [20]. There was also some confusion as to whether such incentives were given to reward good work already done, or work still to be accomplished. The respondents from Ghana and Tanzania described their own motivation as fair to low, which would fit with this understanding - especially given the views expressed about salary levels and the widespread resentment held about access to allowances. Despite the definitions given, at other points in the interviews the concept was also shown to be understood more broadly, with reference made to the motivation generated from appreciation. Given that most of the respondents in all three countries were very clear that the goal of their facility was to serve the community and that community appreciation was widely appreciated there could be scope to further the motivation of staff by reinforcing this sense of the purpose to their work [20].

### Job satisfaction

Across the respondent groups from all three countries, reasonable levels of job satisfaction were revealed. These largely stemmed from intrinsic factors, such as appreciation from the community, or the pride the providers took in their work. The latter was particularly the case in Ghana and ties in with the strong sense of professionalism the respondents conveyed. Amongst the findings, it is notable that those who were from the areas in question and worked there for a long time were especially satisfied. Possibly those who choose to remain at a rural posting cannot be but motivated to do so. This provides a further impulse to invest in the recruitment of local students to train as health workers, and to invest in strategies to retain health workers long term [32].

In all cases the policy level respondents underestimated the satisfaction levels of MNH providers. This may indicate that in their functions at policy level they are on the receiving end of complaints and that this blurs their perceptions. It should be noted that the topic guide focused on job satisfaction and made no reference to dissatisfaction.

None of the respondents reported their superiors taking a specific interest in their satisfaction. Given the many problems these rural health providers described, it would seem that their job satisfaction may be the reason for their continued work effort [38]. This would suggest a need to pay closer attention to, and reinforce, MNH provider satisfaction.

### Working conditions

Across all the responses, MNH workloads were felt to be especially heavy. In general, this was attributed to the nature of the work and the overall shortage of staff. In Ghana this stood at odds with the perception of urban providers that their counterparts in rural areas were lazy - a finding which could again reflect the lack of status accorded to rural health work. Lack of staff at facility level is known to be discouraging and to rob providers of professional exchange and support [39]. In addition, many observed MNH to be an area of occupational risk, with efforts to counter this appreciated [40].

There were indications from Burkina Faso that other providers were reluctant to become too involved in MNH tasks. Despite their low numbers female providers at primary care level in Burkina Faso may carry a disproportionate burden of MNH work, as has been observed in other settings [41]. This could be addressed by revisiting the division of labour across facility teams, with particular attention paid to gender aspects. There could be a need to further incentivise MNH work. However, the difficulties of applying financial incentives in isolation are noted in the literature [42]. For example, in Ghana such a scheme gives midwives 10% of the fee paid for a delivery. The recipients scorned this amount as being derisory. The policy level respondents emphasized how financial incentives need to be in proportion with the recipients basic salary to make a difference.

### Management

Managing rural facilities appeared to be facilitated in Burkina Faso by the pervasive sense of discipline, respect for hierarchy and possibly for patriarchy as most facility and district managers were male. A system of staff appraisal has yet to be fully established. Together these factors probably explain the negligible importance attached to such management tools by the respondents. In Burkina Faso national policy is for biannual integrated supervision to be carried out by district managers [9]. When carried out well respondents described such supervision as very encouraging. However, strong resentment was felt when district supervisors failed to appear or acted in haste, as has also been found in other studies [43].

In Tanzania the role of facility managers appeared to be more difficult. Whilst the existence of a public sector instrument for performance appraisal is undoubtedly positive, its implementation appears to be challenging and it likely that it will take time for its use to become established [20]. Supervision in Tanzania is team-based [7] but difficulties in getting the team together were noted. The predominance of lower level cadres at primary level makes supervision extremely important. Given the difficulty these staff were shown to face in asking for help and the managers’ views about having to allocate tasks to individuals to ensure results, a more supportive, individualized approach to supervision, as exercised in Ghana, could be helpful. Indeed, the respondents from Ghana were the only ones to mention supervision as a non-financial incentive. An annual performance management tool is quite well established in Ghana. However, an effect of this has been that great importance is attached to winning the favour of managers, resulting in competition between staff. The complaints about managers using their positions to avoid the more unpleasant aspects of work showed that this importance is not necessarily linked to respect.

The differing recent histories of the three countries may help to explain the divergent findings with regards facility management. Whilst respect for hierarchies is acknowledged to be strong across Sub-Saharan Africa [44], the French working culture may have served to particularly reinforce this tendency in Burkina Faso [45]. Tanzania, by contrast, experienced a period of socialism, and its people enjoy a common national language, which includes the leveling concept of the ‘ndugu’ or brotherhood. However, Okema (1996) has associated this with challenges including indifference and a disregard for authority [46]. This may help explain the described decline in discipline and the anxiety providers feel at being blamed for issues beyond their control. Chandler et al., 2009 observed that traditional values in Tanzania are currently experiencing a period of erosion [24].

### Remuneration

A comparison of starting monthly salary for a midwife in the three countries show the figure to be approximately 750 UDS in Ghana [47], 180 USD in Burkina Faso [48] and 175 USD in Tanzania [49]. Whilst this measure is limited as it does not include allowances or illustrate the purchasing power parity, it does provide us with an indication.

Against this backdrop it is interesting to note the acceptance of low salary found amongst some of the respondents in Burkina Faso, especially the lower cadres and female providers. It can perhaps be explained by the importance of hierarchies and gender roles. Indeed, others have found the main reason health workers in Burkina Faso remained in the public sector was for the provision of a good salary [50]. However, it also emerged that more experienced and better-qualified providers did not share this acceptance and were more likely to complain about salaries. The district level respondents from Burkina Faso included medical doctors. They all compared their salary to that of neighbouring countries and indicated a readiness to emigrate. This readiness was not reflected amongst the sub-cadres of physicians that make up the other district respondents. This could reflect the limited possibilities that such sub-cadres have to emigrate [51].

The responses from Tanzania and Ghana were unanimously dismissive of salary levels, considering them to be low in relation to the responsibility and volume of work. This is despite the fact that in 2006 Tanzania implemented a salary increase for public sector health workers and Ghana introduced a new public sector wage structure [52]. The high degree of suspicion that is revealed as to who earns what could be overcome with greater transparency. The missing link between salary and performance was noted across all the countries. However, whilst in Burkina Faso it appeared that the notion of ‘moral motivation’ and decorating highly performing staff could help to bridge the gap, in Tanzania and Ghana the gap was shown to be resented. This ties in with the predominant understanding of motivation as something that was given in addition to salary to encourage providers to perform well. Work experience was also not perceived to be recognised in salary levels, which were considered to be tied to qualifications, with token increases for additional functions. This would offer potential to specifically incentivise work experience in rural areas, for example, for it to be better paid or to lead to opportunities for further education or promotion [31,44,53].

In the responses from Ghana there was a very open discussion about the financial importance of being able to undertake additional work in the private sector. Indeed, this may be a further reason that rural placements were so disliked [54]. In Burkina Faso and Tanzania only district level staff mentioned working extra shifts in private facilities, which may reflect that such possibilities are limited for providers based in remote areas.

### Interpersonal relations and quality of care

Appreciation from the community was described as encouraging in all the contexts. However, relations with communities were also shown to be complex. From all the countries the providers indicated that at a rural facility, and especially in the area of MNH, one’s level of competence is readily exposed [55]. This could partly explain why the lower cadres in Tanzania felt unsupported by their managers [20]. From Burkina Faso there were reports of patients seeking to win the favour of specific health workers with gifts and of complaints of sexual harassment when maternal care was provided by men. Of particular concern is the view that communities were to blame for not seeking care in a timely manner, which could be indicative of a wider problem regarding the ethics of health workers. D'Ambruoso et al. [56] describe how interpersonal relations between providers and patients hold the key to quality care [56]. It would seem that some providers need to be specifically encouraged to take personal responsibility for tasks or to work to the best of their ability.

The complaints made about the poor way that ‘other ‘or ‘younger’ providers, providers from different provinces, or simply providers ‘these days’ treated patients could be references to certain tendencies or contradictions in the respondents own character from which they prefer to disassociate themselves. Alternatively, the outrage this poor treatment instills in some of the respondents could indicate a need to afford peer criticism greater attention. It is interesting that the providers’ personal faith was often referred to as a source of strength for not being drawn into such behaviours. It is possible that encouraging greater reflection on ones practice by creating supportive spaces where poor outcomes can be discussed, or where providers are sensitized to empathise with their patients, could also hold some potential.

Only in Burkina Faso were examples given of health workers feeling sufficiently secure in their teams to ask for help. This may be because higher qualified staff were often shown to look down on other providers. Chandler et al. [24] also note the existence of ‘snobbery ‘between highly trained and auxiliary workers in Tanzania [24]. In Ghana competitiveness between staff appears to make it difficult for colleagues to help one another. The lack of acceptance of which staff are selected to win awards may also be a reflection of this. Freitas (2009) describes how it can be very hard for auxiliary staff to ask for help when uncertain and that this makes them particularly likely to hide their mistakes [57]. These findings underline the difficulties that are likely to surround the use of practices such as maternal audits and learning from medical errors.

### Incentives

Overall, there is considerable convergence in the types of financial incentives that were suggested across the three groups of respondents from both the public and private sectors. In Burkina Faso a rural allowance was considered very pressing. This was despite the fact that the housing allowance provided for all civil servants is notably higher in rural areas. Health insurance for health workers was also mentioned. This was already reported to be in place by the respondents from the other countries. The strong call for a reinstatement of the additional hours allowance in the responses from Ghana reflects how the withdrawal of benefits is an enduring cause of resentment [58]. Moreover, the respondents from Ghana expressed particular concerns pertaining to pension provision, which were mirrored by the faith-based respondents from Tanzania. Indeed, the differences in employment conditions between public and facility-based health workers in Tanzania have been shown to have more to do with additional benefits than the salary itself [59].

There is also convergence in the types of non-financial incentives that were proposed. However, there were contrasts too, with supervision only found in the suggestions from Ghana and decorating health workers only mentioned frequently in the responses from Burkina Faso.

Amongst the respondents from Burkina Faso, there was strong agreement that incentives should be given to teams, with the exceptions of prizes for health workers. In Ghana the respondents had mixed views, whilst the responses from Tanzania revealed a preference for incentives to be given to individuals. The strong sense of team in Burkina Faso requires further clarification. It is possible that the Tanzanian respondents preference for incentives to go to individual providers may have been coloured by the ongoing, in-country policy debate and pilot schemes that feature this modality.

### Limitations

This study drew its respondents from rural health centres in two or three districts of the countries concerned. The authors collectively command a strong knowledge of the settings and consider the respondents demonstrate ‘recognizable consistency’ with their context [60]. Given the coherence of the findings, they may be transferrable to other very similar, rural settings in the three countries concerned. Beyond the importance of the findings for the QUALMAT project’s ongoing research, the issues may reflect phenomena of broader relevance, of which policy-makers in the three countries should be cognisant. However, they clearly cannot be generalised statistically. By comparing the findings the tendency to generalisation may have been intensified and the reader is cautioned to this effect.

Furthermore, attention is drawn to a potential selection bias as only MNH providers and managers that were at their posts could be interviewed. It is arguable that these respondents are likely to be comparatively well motivated. Alternatively, they may have remained in their positions due to the failure of policies and management strategies and be less motivated for this very reason. The possible distortion that this may have introduced to the understandings of motivation should be born in mind.

It was retrospectively observed that the fact that the interviewers were part of the same social fabric as the respondents may have blinkered their ability to respond to the emerging definitions. Combined with the reasonably structured nature of the topic guide, this may have resulted in concepts not being fully explored.

## Conclusions

The terms and concepts pertaining to motivation were shown to vary across the three settings. The extent to which this influenced the way providers described and perceived their own levels of motivation requires greater clarification.

In Burkina Faso, provider sex appeared to play a role in rural staff recruitment and available female providers may be carrying a particularly high MNH workload. This underlines the importance of gender-sensitive health workforce planning and retention strategies for progress in MNH.

Across all the countries there were indications that providers sometimes failed to treat their patients well and might find it hard to ask their colleagues for help. The training that all levels of MNH providers receive in professional ethics and the way this training is reinforced in practice, including through supervision, require closer attention.

Given the extent of the contrasts between the findings, in-depth country-level research may be considered an essential precondition for the introduction of incentive schemes. In all three settings, some providers who were willing to work in rural areas were found. Greater effort could be invested in understanding their profile, the types of incentives they would prefer, and if there are modalities that could reinforce this readiness to remain.

Any incentive scheme will be affected by the facility management capacity, the challenges associated with establishing performance appraisal tools, the intransparency shown to surround financial issues in some of the settings, pre-existing divisions between cadres as well as local preferences for certain types and modalities of incentives.

### QUALMAT

The QUALMAT research project (Quality of Maternal and Prenatal Care: Bridging the Know-do Gap) funded as part of the 7th Framework Programme of the European Union (grant agreement 22982) is a collaboration between the Centre de Recherche en Santé de Nouna (Burkina Faso), Ghent University (Belgium), Heidelberg University (Germany), Karolinska Institute (Sweden), Muhimbili University of Health and Allied Sciences (Tanzania), and Navrongo Health Research Centre (Ghana). The overall objective of this research is to improve the motivation and performance of health workers and ultimately the quality of pre-natal and maternal care services. The intervention packages include the development and implementation of a system of performance based incentives and a computer-assisted clinical decision support system (CDSS) based on WHO guidelines. The interventions are evaluated in a pre-post controlled study design in rural Burkina Faso, Ghana and Tanzania between 2009–2014.

## Abbreviations

GNI: Gross National Income; MDG: Millennium Development Goal; MNH: Maternal and neonatal health; OECD: Organisation for Economic Cooperation and Development; PHC: Primary Health Care; USD: American dollars.

## Competing interests

The authors declare that they have no competing interests.

## Authors’ contributions

HP was highly involved in development of the topic guide, undertook preliminary coding for Tanzania data, cross coding and further rounds of coding and analysis for all countries, led process of exchange during analysis, prepared the first draft of the article. MK gave input to the topic guide, undertook the pre-test of the French version of the topic guide, conducted the interviews in Burkina Faso, transcribed the interviews, undertook preliminary coding, endorsed the further stages of coding and analysis for the Burkina Faso data. GA gave input to the topic guide, conducted and transcribed the interviews in Ghana, undertook preliminary coding, endorsed the further stages of coding and analysis for the Ghana data. JW gave input to the topic guide, endorsed the further stages of coding and analysis for the Ghana data. DK gave input to the topic guide, undertook the pre-test and conducted the interviews in Tanzania, transcribed and translated the interviews, coordinated the quality control thereof, endorsed the stages of analysis for the Tanzania data. MTL led the processes of developing the topic guide, endorsed the coding and analysis of the Tanzania data after each round and contributed to the development of the article. MY gave input to the topic guide, endorsed the stages of analysis for the Burkina Faso data, provided input to the article. MM backstopped the development of the topic guide and contributed to the article. RS contributed to the study design, contributed to developing the topic guide, oversaw the analysis and contributed to the article. All the authors read and approved the final manuscript.

## Pre-publication history

The pre-publication history for this paper can be accessed here:

http://www.biomedcentral.com/1472-6963/13/149/prepub
